# Gender differences in the subjective well-being of older adult learners in China

**DOI:** 10.3389/fpsyg.2022.1043420

**Published:** 2022-11-09

**Authors:** Xiaowei Shi, Yuan Li, Lixin Sun, Yong Yu, Shiyu Zhou

**Affiliations:** ^1^College of Teacher Education, Ningbo University, Ningbo, China; ^2^Affiliated School of Ningbo University, Ningbo, China

**Keywords:** older adult learners, subjective well-being, empirical analysis, older adult learning, gender difference

## Abstract

The trend of the feminization of the aging population in China is accelerating, and the differences in the subjective well-being of older adults are becoming more and more obvious. This study combines gender theory with gender differences as an entry point, based on 436 survey data, and examines effects and differences between learning engagement on the subjective well-being of older Chinese learners by gender. The study found the following: learning can enhance the subjective well-being of older people; there were significant differences in the subjective well-being of older adult learners by gender in two dimensions, namely, physical and mental health experience and adaptation satisfaction experience; and the positive effect of learning engagement on subjective well-being of female older adults was more pronounced and thus had a stronger effect on their subjective well-being. In addition, the gender equality of older adults’ learning participation and gender differences in learning needs are further explored based on the gender theory perspective, and this gives suggestions for enhancing older adult learners’ subjective well-being.

## Introduction

In 2021, people residing in China aged 60 and above totaled 260 million approximately, with 190 million aged 65 and above (National Bureau of Statistics of China, 2022). According to the Ministry of Human Resources and Social Security of the People’s Republic of China, this number will exceed 300 million by the “14th Five-Year Plan” (period covering 2021 to 2025), and the aging status will change from mild to moderate. In response, the state is vigorously implementing an “active aging” strategy, of which subjective happiness is an important criterion for measuring whether a country or region has achieved active aging. It is also an important indicator for measuring the satisfaction of the elderly (Jopp and Smith, 2006; Tovel and Carmel, 2014) because a high subjective well-being (SWB) is a result of the continuous satisfaction of real-life needs. Therefore, improving the happiness of older people is not only about their quality of life but also has important implications for China to accelerate the realization of active aging.

There are many factors that affect SWB, and learning is one such important factor (Gerdtham and Johannesson, 2001; Bjørnskov, 2003). In recent years, the State Council of China has clearly highlighted the importance of elderly learning in documents such as the “Opinions of the General Office of the State Council on Promoting the Development of Elderly Services,” “Development Plan for Elderly Education (2016–2020),” and “The 14th Five-Year Plan for the Development of the National Ageing Cause and the Elderly Service System.” The Department of State of China (2022) stated that elderly learning is conducive to meeting the diversified spiritual, cultural, and life needs of the elderly, and it is necessary to promote elderly education. Participation in learning can enhance the SWB of older people by meeting some of their needs. In ancient China, it was believed that women did not need to be talented, but simply obedient to their husbands, so old Chinese females have a lower social status than their male counterparts. Consequently, they suffer from unequal opportunities, gender bias, and low social support in learning activities, which leads to gender differences in the subjective happiness of elderly learners (Yang et al., 2016). As the trend of “feminization of the aging population” becomes increasingly obvious (Jia B. J., 2016), older females have become a large group that cannot be ignored. Elderly females have also increasingly participated in educational activities, suggesting the trend toward active aging, the performance of SWB among the elderly of different sexes also showed obvious differences. Previous studies on the relationship between senior learning and SWB have not yet reached a unified conclusion, and relatively little attention has been paid to the SWB of female seniors. In order to achieve the goal of active aging and improve the overall SWB of seniors, we must pay attention to the gender differences in senior learning and strengthen the study of gender differences among seniors. Based on this, this study investigated the relationship between older people’s learning participation and their SWB to find out the corresponding gender differences and influential factors, starting from the relationship between older adults’ learning participation and SWB, in order to provide a theoretical basis for improving the well-being of older adults and to provide an effective reference for the study of SWB heterogeneity among older adults.

## Theory and literature

### Gender theory

Social gender differs from biological gender in that it is a component of one’s culture (Liu, 2001). It mainly refers to the understanding of the differences between men and women, including female or male group characteristics and the behavioral modes that are formed in the process of social and cultural formation (Tan and Xin, 1995). Gender theory originated from the United States, mainly relating to Western intellectual traditions on the relationship between the sexes’ “naturalistic attitude” and the dichotomy between men and women, and then it rapidly expanded into Europe and eventually worldwide in the 1920s, through its proponents such as Gale, Lubin, and Scott. Gender theory divides human gender into biological gender and social gender. It accepts the natural differences between men and women, with focus on the cultural, economic, and family conditions that lead to social differences between the sexes. It places greater emphasis on the non-biological aspects of gender development, especially the construction process of society relating to gender (Cai, 2002). This theory is a rebuttal to the idea that biological sex differences (i.e., biological differences between men and women) determine gender temperament differences and gender identity.

The formulation of gender theory is a unique contribution of Western feminism; its application has turned the attention of gender studies to the social field, focusing the analysis on the impact of social organizations on gender status differences and the relationship between women relative to men (Cai, 2002). It advocates the study of women’s issues within the structure of gender relations and argues that gender differences can be changed. As its core views, the theory holds that gender is a power relationship, a social construct, an institutional arrangement, and a cultural concept (Li, 2015). Feminist scholars such as Kate Millett, Jermaine Greer, and Genevieve Lloyd have explored the causes of gender differences from different aspects (Millett, 1999; Zhang, 2017). Joe Scott’s analysis of gender in his article “Gender: a useful category in historical analysis” particularly provides a useful perspective for us to examine studies on women based on gender theory (He and Wang, 2008). Currently, gender theory has become an epiphenomenon with critical knowledge, and it has gradually developed into an important analytical category in Western academic research, forming several gender analysis frameworks with good applications. Using such a perspective to examine the gender situation in elderly education and gender differences in the SWB of the elderly will further enrich and develop gender theory.

### The present study

#### Measurement of older people’s participation in learning

Various scales exist for measuring learning participation, and the representative engagement measure currently recognized by academics is the Personal Involvement Inventory Scale proposed by Zaichkowsky (1985). In the early days of research, most scholars focused on the measurement of motivation for learning, such as Amabile et al. (1994), who designed the Working Preference Inventory based on studies related to learning/work engagement. This uses a 30-item scale to measure endogenous and exogenous motivation for participation. Burgess (1971) developed the Reason for Educational Participation Scale based on the Adult Learning Scale, which can be used to measure older people’s participation in learning. Canadian scholars Boshier and Collins (1985) developed the new Education Participation Scale (EPS), which is the world’s most recognized and universally used adult learning questionnaire; it includes elements of social contact, career development, external expectations, and cognitive interests. Fredricks et al. (2004) and Kong (2003) have developed scales for behavioral, affective, and cognitive components of learning engagement. The Adult Learning Scale compiled by Li (2008) is divided into five main dimensions: knowledge seeking, career development, interaction, social service, and performance. In the study of learning participation of the elderly, Lv (2016) opined that from the perspective of successful aging, the measurement of learning participation in the elderly should be conducted multidimensionally from three aspects: physiological, psychological, and social. To examine the effectiveness of elderly learning, Jia H. B. (2016) used qualitative interviews to classify the community learning effectiveness of urban female elderly into physical, psychological, and social dimensions, and to measure the physical health care power, psychological completeness, and social integration power of the elderly participants. Pan and Chen (2006) summarized the Adaptation to Learning Test (AAT) developed by the Japanese Institute of Education and the Diagnostic Learning Test (MAAT) revised under the mentorship of Zhou at East China Normal University. In addition, other researchers have developed the Learning Participation Scale for older adults based on the following three dimensions of active aging: health, participation, and security, to reveal the effects of learning in old age (Huang and Ouyang, 2020). In this study, the EPS scale was adapted, and the scale was adjusted based on actual surveys concerning behavioral, cognitive, and emotional aspects (Kaplan, 1960). The three dimensions of learning atmosphere, learning experience, and learning investment were finally adopted as the main dimensions for measuring older people’s learning participation.

#### Relationship between learning and subjective well-being in old age

Previous studies on the relationship between learning and SWB have not reached a consensus, with the vast majority of scholars agreeing that learning has a positive effect on well-being (Xiang and Yao, 2017), and the positive correlation between the two is a fundamental finding. Chen (2012) divided the mechanism of the effect of education on well-being into direct and indirect effects and stated that learning has a significant positive effect on well-being. Oswald (1997) also demonstrated that residents who participated in learning continued to present a higher SWB even after controlling for other variables. The relationship between older people’s learning and their SWB has also received extensive academic attention in these studies, with some researchers suggesting that participation in learning has a negative impact on well-being, because people’s happiness depends on whether their expectations are met. Specifically, excessive expectations negatively affect people’s well-being, and learning raises people’s subjective expectations to some extent, which has a negative impact on happiness (Clark and Oswald, 1996). Stutzer (2004) and Knight et al. (2010) argued that the negative effect of learning on happiness is influenced by group differences and that participation in learning has a negative impact on the happiness of urban immigrant groups. However, some scholars suggested no correlation or weak significance of the relation between participation in learning and SWB. For example, the study by Ferrer-i-Carbonell’s and Frijters of middle-aged and older adults showed no correlation between learning and SWB (Ferrer-i-Carbonell and Frijters, 2004). Inglehart and Klingemann (2000) suggested that people’s well-being is influenced by a variety of factors and that learning may act as a mediating variable that influences older adults’ SWB. By contrast, Escuder-Mollón et al. (2014) argued that participation in learning activities improves the quality of life of older people and increases their well-being. A study by Fan (2009) also verified a significant positive relationship between learning and SWB in older people. Another study found that older people who participate in learning activity societies have higher SWB than those who participate in general societies (Wan et al., 2006). In exploring the relationship between older people’s participation in learning activities and their SWB, we propose:

*H1*: A positive relationship exists between learning engagement and SWB in older people.

#### Gender differences and factors influencing elderly learners’ subjective well-being

Many researchers (von dem Knesebeck et al., 2003; Burnay et al., 2005; Tang et al., 2006) have analyzed the SWB of older people in terms of demographic, social, child, economic, and personality factors. In their study of gender differences in the SWB of older people, Li et al. (2007a) found that majority of female older people had “general” and “better” SWB, but lower levels of well-being than their male counterparts. These authors found that a high illiteracy rate among older women leads to a lower economic, social, and family status. In addition, a higher life expectancy and proportion of widowhood of women compared with men leads to a higher rate of living alone, which also contributes to the relatively lower SWB scores of older women (Li et al., 2007b). Wang et al. (2015) found from a survey of rural older adults in northern Anhui Province that the SWB of rural female older adults was at an intermediate level, but poorer compared with rural male older adults, which may be caused by family pressure, relationship with mother-in-law, education level, widowhood, and other factors. However, a study by Zebhauser et al. (2014) reached an opposite conclusion—male older adults had lower levels of SWB compared with female older adults. Wang (2015), through a literature review of SWB measured in older adults, also found that although most studies concluded that older women had lower levels of well-being and higher levels of depression than men, female older adults had higher levels of mental health and greater increases in SWB than male older adults when life contexts were basically the same. By contrast, a meta-analysis of 21 papers by Ren et al. (2010) revealed that gender was a low predictor of SWB, indicating that there was no significant difference in the SWB of male and female older adults.

With the popularization of elderly education and the improvement of old-age security programs, elderly people are increasingly participating in learning activities. Studies by different scholars have confirmed the correlation between learning and the SWB of elderly people and have explored the effect of participation in learning on their SWB. However, two deficiencies remain. First, most of the previous studies were based on theoretical research, and less empirical research was conducted on the learning and participation of local elderly people. Second, most studies on SWB were based on older people as a whole, and few studies have differentiated the effect of learning participation on older learners’ SWB from a gender perspective. Studies have shown that the proportion of female learners in older people’s universities was much higher than that of male learners (Shi, 2009) and that the extent and effectiveness of participation in learning activities differed between genders (Fan and Du, 2003). To investigate further whether the SWB of older learners and the effect of learning engagement on the SWB of older learners differ by gender, we propose:

*H2*: Gender differences exist between older learners’ SWB.

*H3*: The positive effect of learning engagement on SWB is more pronounced in female older adults.

## Materials and methods

### Participants and procedures

In this study, a random sampling method was used to select a sample of elderly people aged 50 or above who were involved in elderly education in the subordinate districts and counties of S, a city in the developed southeast coastal region with a high concentration of elderly learners. We developed electronic questionnaires for random distribution through educational institutions such as senior colleges, community colleges, and adult schools, with the consent of older learners. A total of 450 valid questionnaires were collected in 2021. After excluding invalid questionnaires, 436 remained, a return rate of over 96.8%. The final samples consisted of 198 male older adult learners (45.4%) and 238 female older adult learners (54.6%). Table 1 provided the demographic characteristics of the participants in the current study.

**Table 1:** Participants’ demographic characteristics.

	Male	Female	Overall
Total number of individuals	198	238	436
**Age (in years)**			
50–59	73 (16.7%)	87 (20.0%)	160 (36.7%)
60–69	57 (13.1%)	78 (17.9%)	135 (31.0%)
70–79	45 (10.6%)	54 (12.2%)	99 (22.7%)
Over 80	22 (5.0%)	20 (4.6%)	42 (9.6%)
**Marital status **			
Single	57 (13.1%)	60 (13.8%)	117 (26.8%)
Married	141 (32.3%)	178 (40.8%)	319 (73.2%)
**Monthly income **			
Under ¥1,000	45 (10.3%)	53 (12.2%)	98 (22.5%)
¥1,000–¥2,000	53 (12.2%)	61 (14.0%)	114 (26.1%)
¥2,000–¥4,000	87 (20.0%)	102 (23.4%)	189 (43.3%)
Over ¥4,000	13 (3.0%)	22 (5.0%)	35 (8.0%)
**Education level**			
Less than junior middle school	73 (16.7%)	69 (22.0%)	169 (38.8%)
Junior high school	76 (17.4%)	71 (16.3%)	147 (33.7%)
High school (includes technical secondary school and professional high school)	30 (6.9%)	39 (8.9%)	69 (15.8%)
Junior college	13 (3.0%)	19 (4.4%)	32 (7.3%)
College/Bachelor’s degree or above	6 (1.4%)	13 (3.0%)	19 (4.4%)
**Living conditions**			
Living alone	29 (6.7%)	33 (7.6%)	62 (14.2%)
Living with partner only	58 (13.3%)	64 (14.7%)	122 (28.0%)
Living with children	53 (12.2%)	61 (14.0%)	114 (26.1%)
Living with partner and children	45 (10.3%)	64 (14.7%)	109 (25.0%)
Living in a residential care facility	13 (3.0%)	16 (3.7%)	29 (6.7%)

### Variable setting and measurement tools

#### Demographic variables

The questionnaire had three parts. The first asked about demographic variables in two sections: family characteristics such as marital status and residential status, and individual characteristics such as gender, age, education level, and monthly income (Table 2). The results of the SPSS reliability and validity tests revealed that the Cronbach’s alpha value of the whole questionnaire was 0.927, indicating that the reliability and validity of the questionnaire met the requirements of this study.

**Table 2:** Variable codes and explanations.

	Value	Explanation of variables
Age	1–4	1 = 50–59; 2 = 60–69; 3 = 70–79; 4 = over 80
Gender	1–2	1 = Male; 2 = Female
Marital status	1–2	1 = Married; 2 = Single
Living conditions	1–5	1 = Living alone; 2 = Living with partner only; 3 = Living with children; 4 = Living with partner and children; 5 = Living in a residential care facility
Monthly income	1–4	1 = Under ¥1,000; 2 = ¥1,000–¥2,000; 3 = ¥2,000–¥4,000; 4 = Over ¥4,000
Education level	1–5	1 = Less than junior middle school; 2 = Junior high school; 3 = High school (includes technical secondary school and professional high school); 4 = Junior college; 5 = College/Bachelor’s degree or above
**Subjective well-being **		
Physical and mental health experience	1–6	From 1 (strongly disagree) to 6 (strongly agree) The higher the score, the higher the subjective well-being of the older people
Adaptation satisfaction experience		
Self-development experiences		
**Older adult learning **		
Learning investment	1–6	From 1 (strongly disagree) to 6 (strongly agree) The higher the score, the higher the level of participation in learning by older people
Learning atmosphere		
Learning experience		

#### Older adult learning

The independent variable in this study was older adult learning. The scale had three main dimensions: older learners’ learning investment, learning atmosphere, and learning experience. Older learners’ learning investment included learning time investment, the number of courses, and the amount of money invested. Learning atmosphere was mainly measured from three dimensions: teachers’ teaching ability, teacher–student relationship, and the relationships among students. Learning experience was mainly measured from the dimensions of learning interest and learning initiative, challenge difficulty and the importance of learning, and so on. The period of learning assignment method ranged from 1 (less than 1 year) to 4 (more than 10 years), the number of courses ranged from 1 (one) to 4 (more than four), and the cost of education ranged from 1 (0 yuan) to 5 (more than 200 yuan per month). The other variables were scored on a six-point scale with values ranging from 1 (strongly disagree) to 6 (strongly agree)—the higher the score, the better the educational experience. In the data computation, because of the different scales used for the independent variable, the data were standardized to avoid large arithmetic errors. The results of the questionnaire reliability test showed that the Cronbach’s alpha value for the total scale was between 0.80 and 0.93, indicating that the scale met the requirements of this study.

#### Subjective well-being

The dependent variable of this study was SWB, which included three measurement dimensions: physical and mental health experience, adaptation satisfaction experience, and self-development experience. The research scale was based on the Subjective Well-being Scale for Chinese Citizens (SWBS-cc) (Xing, 2008) and was adjusted according to the research needs. The physical and mental health experience was measured from the experience of physical health, mental health, and mental balance. The self-development experience was measured from the experience of growth and progress, target value, and self-acceptance. The experience of adaptation and satisfaction was measured from the experience of family atmosphere, interpersonal adaptation, contentment, and social confidence. The scale comprised 10 items rated on a six-point scale, ranging from 1 (strongly disagree) to 6 (strongly agree), with higher scores indicating higher SWB of older learners. The Cronbach’s alpha value was 0.886, indicating good overall validity and reliability, and statistical significance.

### Data analyses

We used SPSS 26.0 for the analysis. First, descriptive statistical analysis and difference test were used to analyze the differences in the SWB of older adult learners and the dimensions of older adult learning in terms of demographic variables. Second, correlation analysis was performed to verify whether older adult learning (learning investment, learning experience, and learning atmosphere) affected the older adult learners’ SWB. Third, independent t-test was done to verify whether a significant difference existed in the SWB of male and female older learners. Finally, stratified regression was performed to examine the mechanism of older adult learning on the SWB of male and female older learners and to discover the differences in the effect of older adult learning on enhancing the SWB of male and female older adults.

## Results

### Descriptive statistical analysis and analysis of variances

According to the research data (Table 3) on the SWB dimension, significant differences existed in the SWB of older learners with different marital status, and single individuals were significantly happier than married ones. The same was true for the income level dimension—those with disposable income of RMB 2,000–4,000 had the highest SWB and those with less than RMB 1,000 had the lowest SWB. Significant differences also existed in the SWB of older learners based on living conditions, while no significant difference was found based on the education level. For the older learning participation dimension, there was no significant difference in the learning atmosphere, learning experience, and learning investment of older learners with different marital status, age, and education level. However, significant differences were found in two aspects, namely monthly income and living conditions. Older learners with disposable income of RMB 2,000–4,000 had the best learning atmosphere, learning experience, and the most learning investment.

**Table 3:** Descriptive analysis.

	SWB	Learning atmosphere	Learning experience	Learning investment
Married	−0.05 ± 0.82	−0.026 ± 1.00	0.03 ± 1.02	0.01 ± 1.02
Single	0.14 ± 0.69	0.07 ± 1.01	−0.09 ± 0.93	−0.02 ± 0.96
*t*	−2.22^*^	−0.89	1.16	0.28
50–59	−0.15 ± 0.79	−0.07 ± 0.95	−0.11 ± 1.01	0.02 ± 1.03
60–69	0.14 ± 0.82	0.16 ± 1.02	0.15 ± 1.02	0.06 ± 1.05
70–79	0.07 ± 0.78	0.07 ± 0.99	0.01 ± 0.99	−0.12 ± 0.87
Over 80	−0.03 ± 0.64	−0.41 ± 1.04	−0.07 ± 0.89	0.04 ± 1.00
*F*	3.76	4.06	1.67	0.68
Under ¥1,000	−0.44 ± 0.65	−0.54 ± 0.95	−0.62 ± 0.92	−0.27 ± 0.89
¥1,000–¥2,000	−0.04 ± 0.77	−0.07 ± 1.02	−0.01 ± 0.98	−0.09 ± 0.98
¥2,000–¥4,000	0.24 ± 0.77	0.38 ± 0.84	0.31 ± 0.92	0.21 ± 1.03
Over ¥4,000	0.07 ± 0.87	−0.29 ± 1.06	0.10 ± 0.89	−0.07 ± 0.99
*F*	18.19^**^	22.89^**^	21.66^**^	5.68^**^
Less than junior middle school	−0.12 ± 0.79	−0.06 ± 1.03	0.04 ± 0.96	−0.00 ± 0.97
Junior high school	0.06 ± 0.82	0.08 ± 0.97	0.01 ± 1.06	0.08 ± 1.02
High school (includes technical secondary school and professional high school)	0.17 ± 0.78	−0.04 ± 1.00	0.06 ± 0.93	−0.10 ± 1.11
Junior college	0.14 ± 0.66	0.13 ± 1.00	−0.13 ± 0.94	0.03 ± 0.84
College/Bachelor’s degree or above	−0.21 ± 0.72	−0.15 ± 0.95	−0.40 ± 1.15	−0.30 ± 0.94
*F*	2.50	0.62	1.03	0.87
Living alone	−0.25 ± 0.72	−0.27 ± 1.02	−0.16 ± 0.95	−0.05 ± 1.04
Living with partner only	0.25 ± 0.83	0.33 ± 0.99	0.38 ± 0.87	0.22 ± 1.06
Living with children	−0.25 ± 0.70	−0.37 ± 0.92	−0.45 ± 0.98	−0.20 ± 0.92
Living with partner and children	0.17 ± 0.79	0.17 ± 0.93	0.13 ± 1.00	0.09 ± 1.01
Living in a residential care facility	−0.16 ± 0.68	−0.02 ± 1.00	0.01 ± 0.99	−0.37 ± 0.65
*F*	9.49^**^	10.00^**^	12.37^**^	4.00^*^

*p ≤ 0.05; **p ≤ 0.01; ***p ≤ 0.001.

### Correlation analysis of senior learning and subjective well-being

To identify the relationship between learning participation and SWB of older people, a Pearson correlation analysis was conducted on older people who participated in learning activities after case selection of the original data in the SPSS software (Table 4). The results showed that all dimensions of older people’s learning participation were positively correlated with physical and mental health experience, self-development experience, adaptation satisfaction experience, and overall SWB. Moreover, the correlation was significant (*p* < 0.01), suggesting that older people’s participation in learning activities can significantly enhance their SWB. Therefore, Hypothesis 1 is supported.

**Table 4:** Correlation between variables.

	1	2	3	4	5	6	7
Learning atmosphere	1						
Learning experience	0.320^**^	1					
Learning investment	0.278^**^	0.356^**^	1				
Physical and mental health experiences	0.439^**^	0.530^**^	0.371^**^	1			
Self-development experiences	0.443^**^	0.480^**^	0.424^**^	0.433^**^	1		
Adaptation to meet the experience	0.440^**^	0.508^**^	0.382^**^	0.439^**^	0.454^**^	1	
Subjective well-being	0.556^**^	0.639^**^	0.496^**^	0.787^**^	0.794^**^	0.796^**^	1

*p ≤ 0.05; **p ≤ 0.01; ***p ≤ 0.001.

### Independent t-test of gender differences In older learners’ subjective well-being

To clarify further the gender differences in the SWB of older learners, a t-test of independence was conducted on the sample data in SPSS (Table 5). Significant differences were found in the experience of physical and mental health and adaptation satisfaction experience among older learners of different genders. In terms of mean values, the experience of physical and mental health, experience of self-development, and adaptation satisfaction experience were higher for female older learners than for male older learners. In addition, significant differences existed in the learning investment between genders, with female older learners having higher mean values than male older learners in all dimensions of learning investment. Overall, significant differences were found in the level of learning participation and SWB of older learners by gender. Therefore, Hypothesis 2 is supported.

**Table 5:** Results of independent-samples t-test (*n* = 436).

Variables	Male	Female	t	p
	M (SD)	M (SD)		
Older adult learning				
Learning investment	−0.10 (0.93)	0.08 (1.05)	−1.97	0.05^*^
Learning atmosphere	−0.06 (1.01)	0.05 (0.99)	−1.15	0.25
Learning experience	−0.09 (0.93)	0.08 (1.05)	−1.78	0.08
SWB				
Physical and mental health experience	−0.13 (0.95)	0.11 (1.03)	−2.46	0.01^**^
Self-development experiences	−0.08 (0.98)	0.07 (1.02)	−1.52	0.13
Adaptation to meet the experience	−0.15 (0.90)	0.13 (1.06)	−2.96	0.00^***^

^*^p ≤ 0.05; ^**^p ≤ 0.01; ^***^p ≤ 0.001.

### Stratified regression analysis

To explain further the effect of learning participation on the SWB of older people by gender, this study adopted a stepwise regression method, based on the abovementioned descriptive statistical and correlation analyses, and conducted stepwise linear regressions for all, male, and female older adults. Model 1 only included learning variables (learning atmosphere, learning experience, and learning investment). In Model 2, individual characteristics of the demographic variables (age, education level, and monthly income) were added. Model 3 included the factors of family demographic variables (availability of spouse and living conditions) to reflect more comprehensively the factors influencing the SWB of older learners of different genders and the changing trends. Y was the dependent variable (SWB); *X*1, *X*2, *X*k were the independent variables (older learners and their factors), and the stratified regression model was set as follows (Table 6):


(1) Yi=β0+β1X1+β2X3⋯+βiXi+ε

The regression analysis results of the overall sample of older learners revealed that the standardized equations for the three stratified regressions for the full, male, and female samples were:

Y (full sample) = 0.275 × learning atmosphere + 0.35 × learning experience + 0.192 × learning investment;
Y (male sample) = 0.238 × learning atmosphere + 0.299 × learning experience + 0.203 × learning investment; and
Y (female sample) = 0.308 × learning atmosphere + 0.367 × learning experience + 0.157 × learning investment.

**Table 6:** Regression model for the subjective well-being of male and female older adults.

	Full sample			Male sample			Female sample		
	Model 1	Model 1a	Model 1b	Model 2	Model 2a	Model 2b	Model 3	Model 3a	Model 3b
Learning atmosphere	0.275^***^ (10.569)	0.269^***^ (10.422)	0.261^***^ (10.248)	0.238^***^ (6.832)	0.233^***^ (6.922)	0.216^***^ (6.553)	0.308^***^ (8.068)	0.301^***^ (7.735)	0.299^***^ (7.788)
Learning experience	0.35^***^ (13.059)	0.344^***^ (12.769)	0.352^***^ (13.252)	0.311^***^ (7.885)	0.299^***^ (7.631)	0.30^***^ (7.885)	0.367^***^ (10.087)	0.359^***^ (9.675)	0.372^***^ (10.091)
Learning investment	0.192^***^ (7.252)	0.197^***^ (7.639)	0.200^***^ (7.871)	0.203^***^ (5.220)	0.201^***^ (5.350)	0.211^***^ (5.753)	0.175^***^ (4.865)	0.188^***^ (5.306)	0.186^***^ (5.312)
Age		0.06^**^ (3.334)	0.073^**^ (3.088)		0.065^*^ (2.016)	0.057 (1.804)		0.099^**^ (2.809)	0.090^*^ (2.578)
Monthly income		0.034 (1.247)	0.035 (3.088)		0.028^***^ (0.735)	0.028 (0.771)		0.036 (0.917)	0.036 (0.926)
Educational level		0.084^***^ (3.924)	0.086^***^ (4.066)		0.123 (3.873)	0.129^***^ (4.181)		0.060^*^ (2.072)	0.058^*^ (2.008)
Marital status			0.206^***^ (3.909)			0.222^**^ (3.149)			0.227^**^ (2.948)
Living condition			0.031 (1.516)			0.054 (1.922)			0.007 (0.260)
*N*	436	436	436	198	198	198	238	238	238
*R* ^2^	0.592	0.613	0.627	0.540	0.574	0.599	0.616	0.631	0.642
*F*	211.65^***^	115.963^***^	92.326^***^	78.004^***^	45.255^***^	37.761^***^	127.962^***^	68.554^***^	54.031^***^

*p ≤ 0.05; **p ≤ 0.01; ***p ≤ 0.001.

The results of Model 1 showed significant differences in the effects of learning to participate on the SWB of older people. In particular, the learning atmosphere, learning experience, and learning investment had a significant positive effect on the SWB of older adults (*F* = 211.650, *p* < 0.001, *R*^2^ = 0.592), which explained 59.2% of the variance probability of SWB. In general, a comparison of the regression results for SWB for the male and female samples showed that the positive effect of two influential factors—learning atmosphere and learning experience—on the SWB of female older adults was higher than that of male older adults. These results indicate that learning atmosphere and learning experience had a higher effect on the SWB of female older adults, and only one factor, learning investment, had a higher effect on the SWB of male older adults than that of female older adults. Overall, the positive effect of learning to participate on SWB was higher for female older adults than for male older adults.

To test the hypothesis further, the variables related to individual characteristics and family factors were added to Models 2 and 3 one at a time. The results showed that the influence of learning investment and learning experience on the overall SWB of the elderly increased, while the influence of learning atmosphere on the SWB of the elderly decreased but remained significantly positive. In the male sample, all dimensions of engagement in learning had a reduced effect on SWB with the addition of individual factors. With the addition of family factors, the effect of learning investment on their influence increased significantly. The regression results for the SWB of the female sample showed that the effect of learning atmosphere and learning experience on their SWB decreased, while the effect of learning investment increased after the addition of individual factors. In addition, the effect of learning experience and learning investment on their SWB increased significantly after the addition of family factors, while the effect of learning atmosphere still decreased. Although the effect of learning to participate on SWB changed in the male and female samples after the inclusion of individual characteristics and family factor variables, the positive effects of these two factors—learning atmosphere and learning experience—on the SWB of female older adults were still higher than those of male older adults.

Through stratified regression analysis, before and after adding individual and family factors, the positive influence effects of learning atmosphere, learning experience, and learning investment on the SWB of older people of different genders were more obvious and explained more about their SWB. Moreover, the coefficients of learning atmosphere and learning experience on the SWB of female older adults were higher than those of male older adults, indicating that the positive influence of learning to participate on the SWB of female older adults was more obvious. Therefore, Hypothesis 3 is verified.

## Discussion and conclusions

### Discussion

First, in this study, learning enhanced the SWB of older people. Specifically, the learning atmosphere, learning experience, and learning investment all played a positive role in the SWB of older adult learners. These results are consistent with the findings of earlier studies on older learners. For example, Hammond (2004) conducted an interview study of 145 older adults of different ages, genders, ethnicities, and educational levels, and the results showed that the older adults’ participation in formal schools had a positive effect on their mental health, self-esteem, self-efficacy, sense of purpose in life, socialization, and competency. Boulton-Lewis et al. (2006) explored the importance of learning in the process of active aging and confirmed that learning was positively associated with older people’s work, social relationships, mental outlook, emotional conditions, health conditions, and so on, and in particular, significantly positively associated with health status. Regarding the relationship between elderly education and older people’s life satisfaction, a study by Gao (2005) showed that education influenced the increase of older people’s life satisfaction. A study by Sun and Liu (2020) also showed that the SWB of older people who participated in educational activities was higher, and that good education had a positive effect on the SWB of older people.

In addition, individual characteristics and family factors can affect the SWB of older adult learners, with monthly income and marital status having a significant effect on their SWB. First, the SWB of older learners was the highest for those with monthly income of RMB 2,000–4,000, but lower for those with monthly income above RMB 4,000. Social satisfaction theory suggests that good economic conditions can help members of society to have more resources and opportunities to have higher levels of SWB, but this effect is no longer evident once monthly income reaches a certain level (Easterlin, 2001). Second, the SWB of single older learners was higher than that of married older learners, because older learners with spouses focused more on their families; hence, the opportunity to participate in social activities was greatly reduced. Meanwhile, older learners without spouses were more inclined to participate in learning activities, and their participation in learning helped reduce their negative emotions, increased their enjoyment of life, and improved their SWB (Antonucci et al., 2002). The effect of education level on older learners’ SWB was not significant, and there was no uniform conclusion in the literature about the effect of this factor on SWB. Wang (2011) and Gao et al. (2017) showed that older people with a higher education level had higher SWB, while some scholars argued that the association between education level and SWB was no longer significant as the age increased (Yang, 2008).

Second, significant gender differences were found in the SWB of older adult learners in two dimensions: the experience of physical and mental health and the experience of adaptive satisfaction. Gender theory holds that the differences between men and women are, to some extent, socially and culturally constructed (Scott, 1997) and are closely related to the cultural rules and practices surrounding the genders. Owing to differences in the learning atmosphere, learning experience, and learning investment of older learners of different genders, significant differences in their SWB performance are expected, and in line with the study of Hu et al. (2013). Yuan and Li (2017) also pointed out that the complementary functions and happiness provided by marriage were more important to women than to men. In previous studies, female older learners had poorer physical and mental health experiences, cognitive-emotional experiences, and lower SWB than male older learners because of their lower economic, social, and family status or smaller life circles (Li et al., 2007b). With the promotion of education equity and lifelong education, women were gradually detached from the role of a complicated family caregiver, and through their participation in learning activities, they were able to relax physically and mentally. Their spiritual needs were continuously met, such that their experience levels of physical and mental health and adaptation satisfaction gradually became higher than those of male older learners, and their SWB and life satisfaction indices thereby increased.

Third, the positive effect of learning to participate on SWB was more pronounced among female older adults, suggesting that engagement in learning had a stronger effect on enhancing SWB among female older adults. This was in line with the findings of Zebhauser et al. (2014), but differed from the findings of previous studies. In previous studies of gender differences in SWB in older adults, most scholars believed that the SWB of older women was lower than that of older men. Among them, the study of Chen Tianyong et al. showed that the female elderly has a high rate of illiteracy, so the economic, social and family status is low; the population life expectancy is longer than men, the proportion of widowed and the rate of living alone is higher, all these factors lead to the relatively low subjective happiness score of the female elderly.This paper verified that after participating in learning activities, women’s SWB was significantly improved, and even the happiness performance in terms of learning atmosphere and learning experience exceeded that of male elderly people. This can be explained below: First, in China, women have always been taking on the role of caregiver in the family and have been less socially involved. Their social participation is low, and the original “circle of friends” of the elderly women after retirement has been narrowed; thus, when compared with men, most women have the desire to reconstruct their social and recreational circles. Therefore, by participating in educational activities and entering the learning arena, women in their later years can further expand their social circle, adjust or change their previous lifestyle, and enhance their SWB. Furthermore, gender theory suggests that gender role traits have a greater impact on women than men, and that male older people, owing to their own social role expectations, do not usually express their inner feelings directly during learning activities, particularly their negative emotions. Female older adults are more likely to express their negative emotions in learning activities with friends, allowing for the development and maintenance of close partnerships and a greater sense of well-being. For example, Pak (2017) suggested that participation in education can enhance women’s status and improve their quality of life and well-being by changing the traditional family model of raising children for old age. From the perspective of social support, Shumaker and Brownell (1984) further demonstrated that female older adults who engage in learning activities are more likely to have higher SWB than male older adults by making new friends to receive more emotional support (Tao et al., 2019).

Fourth, there were two other aspects for exploring older people’s learning based on the gender theory perspective: the first was gender equity in older people’s learning participation. Gender inequality, as a form of injustice to most concepts of equity in the context of happiness indices and equity satisfaction perspectives reduced public well-being (Klasen, 1999). Because of historical and cultural traditions, gender preferences in learning investment and learning choices have long persisted, and male older people generally had higher opportunities and levels of learning to participate than females older people did. The differences in learning engagement opportunities between the sexes at all levels of education have converged as the country advances in female education, and the participation rate of female older people in learning has been increasing significantly after their retirement. As the findings of this study showed, female older learners were more engaged in learning than male older adults, and female older adults were significantly more motivated to participate in learning. In addition, relevant studies have further demonstrated the value and significance of female older people’s participation in learning. The increased participation in older people’s learning activities and their return to education directly enabled the exploitation of the human resources and labor value of female older people, thus further promoting harmonious social development (Li and Dong, 2022), which to a certain extent alleviated the gender bias in older people’s learning participation. The second issue was the gender difference in older people’s learning needs. According to gender theory, disciplinary constructs and gender distinctions have a mutually generative point of relationship, and the basic principles of disciplinary establishment define which temperament a discipline is associated with in human beings (Braun, 2014). In China, the stereotype of the traditional division of roles within the family, where “Men are in charge; women are not in charge,” has led many male older people to study in senior schools after retirement to play a part in the re-employment of the talent market of older people; therefore, male older learners have a greater demand for “active aging” vocational skills courses. However, as most of the courses offered by senior colleges are leisure and entertainment courses such as calligraphy, art, and life skills, these courses are more attractive to female elderly learners than male elderly learners; thus, female older learners who chose to participate in learning activities were more motivated than male.

Finally, given the importance of close relationships for older adult learning and SWB, more efforts should be made to address the inequalities. This paper proposes the following strategies for learning activities of male and female older adult learners and the improvement of their SWB: (1) Adhere to the gender equality concept and promote equality in education for older people of different genders; (2) Based on programs on sex-specific differences, identify the learning needs of gender-specific older people; (3) Focus on the quality of senior education as the core, making every effort to enrich the learning experience of older people; (4) Make the supply of educational resources precisely focusing on improving learning effectiveness for older adult. These findings have significant implications for the development of active aging and may offer useful guidance for professional counseling for Chinese older adult learners.

### Limitations and future directions

This study explored the relationship between older people’s participation in learning activities and SWB based on gender differences subject to certain limitations. First, the sample was limited to the developed southeastern coastal region, where the development of elderly education is better, and the extensiveness of the data was slightly inadequate. Second, this study only used the results of one survey and did not properly consider the issue of stability. Although the evaluation of a subjective sense of well-being is expected to be relatively stable over time, the subjective psychology of individuals fluctuates; therefore, their subjective sense of well-being will also change. Future research could expand the scope of the sample and the number of surveys conducted to make the data be more representative and to allow a comparison of the relationship between learning participation and the SWB of older people in different regions. This way, the effect of learning participation on SWB of older people could be analyzed more dynamically.

### Concluding remarks

The uniqueness of this study was that, first, previous studies have focused on the influence of objective factors on older people’s SWB, such as social support, the Internet, and socio-economic status (Cui, 2014; Deng, 2021; Wang, 2021; Ye and Li, 2022); few studies have analyzed the influence on older people’s SWB from the perspective of older people’s learning participation; and this present study provided a new perspective on the differences between older people. Second, this paper focused on the gender differences in older learners’ SWB and analyses the effect of learning engagement on older people’s SWB by differentiating between genders, in order to provide a basis for promoting equity in older people’s education and improving older people’s SWB.

The significance of this study is that from a theoretical perspective, it is a refinement of adult learning research and will also enrich the development of gender theory, which is an important guide for subsequent research on the heterogeneity of older people’s SWB. On a practical level, it will not only help government departments to focus on gender differences in older adult’s learning participation and SWB in policy formulation and institutional design, improving the construction of elderly care and elderly education service systems, providing some new basis for the development of elderly education institutions, but also draw the attention of different groups in society to the SWB of older people, and work together for the construction of a learning society and the realization of the goal of active aging.

## Data availability statement

The original contributions presented in the study are included in the article/Supplementary material, further inquiries can be directed to the corresponding author.

## Author contributions

All authors listed have made a substantial, direct, and intellectual contribution to the work and have approved it for publication. 

## Funding

This study was funded by the 2018 General Project of Education “Happiness Measurement and Empirical Research on the Elderly from the Perspective of Education Premium” (No. BKA180235) of the National Social Science Foundation of China.

## Conflict of interest

The authors declare that the research was conducted in the absence of any commercial or financial relationships that could be construed as a potential conflict of interest.

## Publisher’s note

All claims expressed in this article are solely those of the authors and do not necessarily represent those of their affiliated organizations, or those of the publisher, the editors and the reviewers. Any product that may be evaluated in this article, or claim that may be made by its manufacturer, is not guaranteed or endorsed by the publisher.
